# Methodological procedures followed in a school-and community-based intervention to prevent type 2 diabetes in vulnerable families across Europe: the Feel4Diabetes-study

**DOI:** 10.1186/s12902-019-0470-4

**Published:** 2020-03-12

**Authors:** Stavros Liatis, on behalf of the Feel4Diabetes-study

**Affiliations:** First Department of Propaedeutic Internal Medicine, Medical School, National and Kapodistrian University of Athens, Ag. Thoma 17, 11527 Athens, Greece

## Abstract

Feel4Diabetes (standing for: Families across Europe following a hEalthy Lifestyle for Diabetes prevention, http://feel4diabetes-study.eu/) is a school and community based intervention program, aiming to prevent type 2 diabetes (T2D) among families from vulnerable population groups, in six European countries, by promoting healthy lifestyle. In the current issue of BMC Endocrine Disorders, three reviews and three papers providing a detailed description of the methodology used to obtain measurements related to the trial conduction, as well as two papers using original data collected in the Feel4Diabetes-study are presented.

## Introduction

Feel4Diabetes (standing for: Families across Europe following a hEalthy Lifestyle for Diabetes prevention, http://feel4Diabetes-study.eu/) is a school and community based intervention program, aiming to prevent type 2 diabetes (T2D) among families from vulnerable population groups in six European countries by promoting healthy lifestyle.

**Fig. 1:**
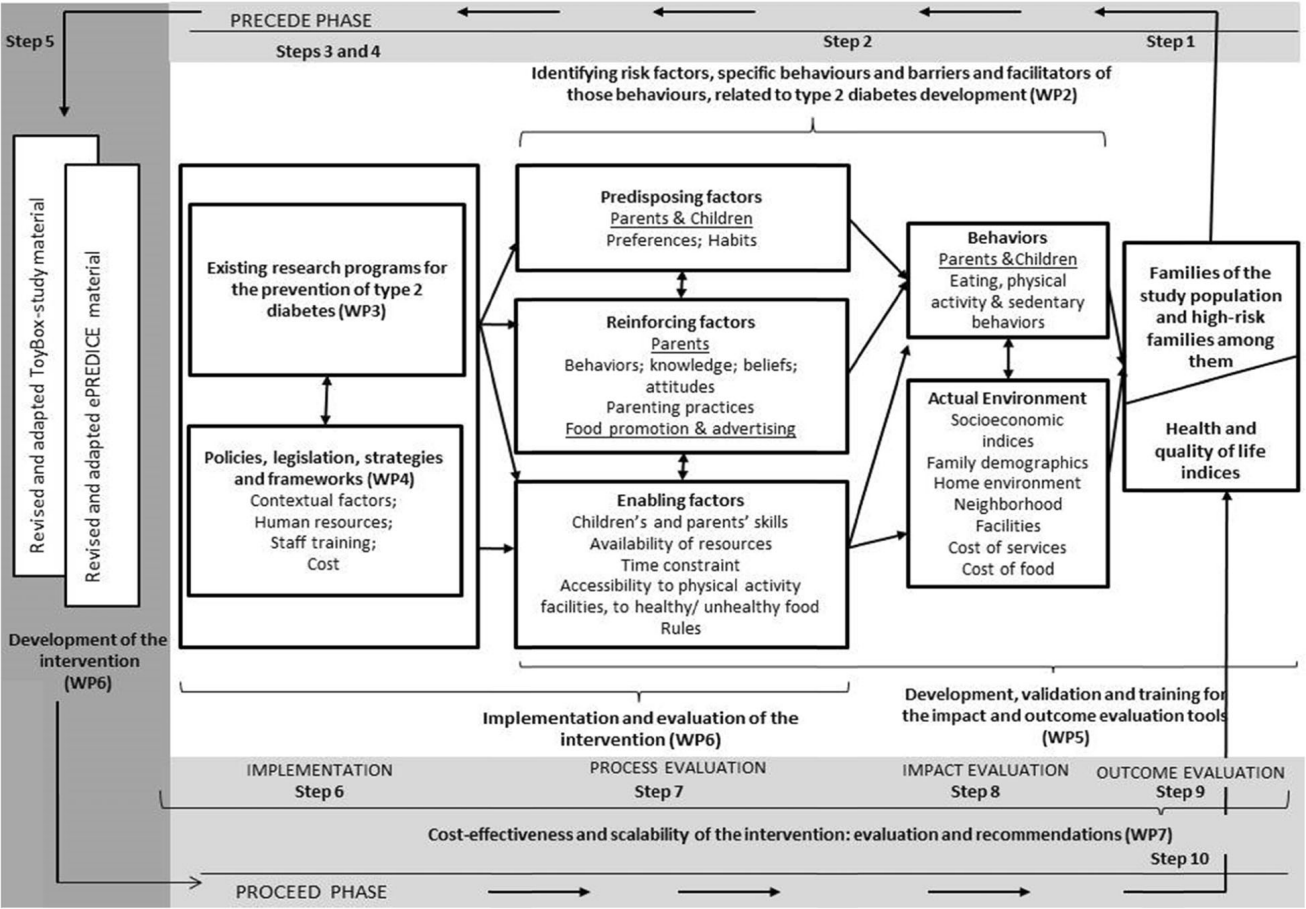
The contextual framework of the Feel4Diabetes project

Participants were recruited from low-socioeconomic areas in high-income countries (Belgium, Finland) or countries under austerity measures (Greece, Spain) and from the overall population in low/middle-income countries (Bulgaria, Hungary). Participating families were recruited from randomly selected schools, clustered within randomly selected municipalities which were randomized to an intervention and a control group. A multidisciplinary team of researchers from seven countries developed the Feel4Diabetes-intervention plan, based on the PRECEDE-PROCEED model and the Health Action Process Approach (HAPA) (Fig. 1) [1]. The Feel4Diabetes-study adheres to the Declaration of Helsinki and the conventions of the Council of Europe on human rights and biomedicine.

The 2-year intervention [2] had two components: the “all families” component, which was delivered at the school setting and the “high-risk families” component which was delivered out of the school, only in families at increased risk to develop T2D, calculated by the validated FINDRISK questionnaire [3, 4]. A “high-risk family”, was identified when at least one parent scored above a country-specific cut-off point for the FINDRISK questionnaire.

The *“all families” intervention component* aimed mainly at the school, home and local municipality environment to assist the family to adopt a healthy and active lifestyle by increasing water consumption (instead of sugary drinks), increasing fruit and vegetables, consuming healthy and balanced breakfast and/or morning snack, increasing physical activity and decreasing/interrupting prolonged sedentary time. The *“high-risk families” intervention component* was additionally implemented to further support and encourage high-risk families to achieve and adhere to the recommendations for a healthy and active lifestyle.

In the current issue of BMC Endocrine Disorders, three reviews related to existing evidence and context of T2D prevention, three papers providing a detailed description of the methodology used to obtain measurements related to the trial implementation, as well as two papers using original data collected in the Feel4Diabetes-study are presented. More specifically: Kyrou et al., present a narrative review on socio-economic factors and key behaviors related to T2D risk. A review of previous research programs implemented in school settings and aiming to prevent T2D is provided by Lambrinou et al. The review by Kivelä et al., aims to outline the existing research and implementation programs that have focused on the identification of adults from vulnerable population groups and on interventions for preventing T2D. The standardization procedure used for collecting valid anthropometric and blood pressure measurements is described in detail by Androutsos et al., while the development, validation and the assessment of the reliability of the questionnaires used in the Feel4Diabetes-study is outlined by Anastasiou et al. A detailed methodology of the cost-effectiveness analysis is described in the paper by Willems et al. In the last two articles, Manios et al., describe the development and testing of a screening procedure to identify individuals and families at high-risk for T2D and hypertension in community settings, while Virtanen et al., aimed to develop a scoring system based on information collected from dietary questionnaires in order to identify families at high-risk to develop diabetes.

Hoping that the outcomes of the Feel4Diabetes-study will provide some new insights and further expand our knowledge and understanding on several aspects of type 2 diabetes prevention, I would like to congratulate the entire Feel4Diabetes-study team for their original and systematic work and acknowledge the essential contribution of school headmasters, teachers, Mayors and city council members. Last but not least I wish to express sincere thanks to the dedicated participants, parents and children for engaging in our study and assist with this important research.

## Data Availability

Not applicable to this introductory commentary manuscript.

## Abbreviation

T2D: Type 2 Diabetes
